# Pleiotropic Effects of Glucocorticoids on the Immune System in Circadian Rhythm and Stress

**DOI:** 10.3389/fimmu.2021.706951

**Published:** 2021-10-08

**Authors:** Akihiro Shimba, Aki Ejima, Koichi Ikuta

**Affiliations:** ^1^ Laboratory of Immune Regulation, Department of Virus Research, Institute for Frontier Life and Medical Sciences, Kyoto University, Kyoto, Japan; ^2^ Department of Human Health Sciences, Graduate School of Medicine, Kyoto University, Kyoto, Japan; ^3^ Graduate School of Biostudies, Kyoto University, Kyoto, Japan

**Keywords:** glucocorticoids, circadian rhythm, stress, cell-mediated immunity, IL-7 receptor

## Abstract

Glucocorticoids (GCs) are a class of steroid hormones secreted from the adrenal cortex. Their production is controlled by circadian rhythm and stress, the latter of which includes physical restraint, hunger, and inflammation. Importantly, GCs have various effects on immunity, metabolism, and cognition, including pleiotropic effects on the immune system. In general, GCs have strong anti-inflammatory and immunosuppressive effects. Indeed, they suppress inflammatory cytokine expression and cell-mediated immunity, leading to increased risks of some infections. However, recent studies have shown that endogenous GCs induced by the diurnal cycle and dietary restriction enhance immune responses against some infections by promoting the survival, redistribution, and response of T and B cells *via* cytokine and chemokine receptors. Furthermore, although GCs are reported to reduce expression of Th2 cytokines, GCs enhance type 2 immunity and IL-17-associated immunity in some stress conditions. Taken together, GCs have both immunoenhancing and immunosuppressive effects on the immune system.

## Introduction

Glucocorticoids (GCs) are a class of steroid hormones with multiple functions. GCs not only regulate functions of the brain, liver, muscle, and bone, they also exert immunoregulatory effects (1). In general, they have strong anti-inflammatory and immunosuppressive effects and are commonly used to treat allergies, autoimmunity conditions, and inflammation by suppressing the expression of inflammatory cytokines, increasing immunosuppressive proteins, and inducing the apoptosis of lymphocytes (1–3). Especially, GCs strongly inhibit cell-mediated immune responses against cancer and infection (4–6). However, recent studies have reported that GCs, when driven by the diurnal cycle or dietary restriction (DR), enhance immune responses by inducing lymphocyte homing to the lymphoid organs (7, 8). Furthermore, stress-induced GCs have the potential to aggravate inflammation by promoting the differentiation and function of Th17 cells (9, 10). Thus, GCs play important roles both in immune responses against infection and cancer and in triggering inflammation. In this review, we will discuss the immunoenhancing and immunosuppressive functions of GCs, which depend on the immune microenvironment.

## Production and Action of GCs

In steady state, GC production is controlled by circadian rhythm through multiple steps (1). First, the suprachiasmatic nucleus (SCN) stimulate the paraventricular nucleus (PVN) of the hypothalamus, which secretes corticotropin-releasing hormone (CRH). The circadian rhythm of the SCN is regulated by transcriptional-translational feedback loop (TTFL) of the molecular circadian clock comprising positive, negative, and accessory loops (11). In addition, light input from retina controls the TTFL in the SCN to synchronize the intrinsic circadian rhythm with environmental light/dark cycle. Next, CRH goes on to stimulate the anterior pituitary to produce adrenocorticotropic hormone (ACTH) into the blood. ACTH induces the expression of the enzyme 11β-hydroxylase, which catalyzes the synthesis of corticosterone and cortisol in the adrenal cortex. Lastly, secreted GCs suppress CRH production from PVN by negative feedback (12). Due to the induction and suppression of GC production, serum GC levels exhibit diurnal oscillation, with a peak at early morning and a nadir at night in diurnal animals like humans, but the opposite in nocturnal animals like rodents. Moreover, psychological, physical, and nutritional stresses induce high levels of GCs. Adrenergic neurons in the locus coeruleus (LC) of the brain stem sense the stress and produce noradrenaline to stimulate CRH-releasing neurons (13, 14). In addition to neuronal signals, inflammatory cytokines such as IL-1, IL-2, IL6, IL-12, TNF-α, and IFN-γ stimulate the hypothalamus-PVN axis and induce GC production (15).

After the production of GCs from the adrenal cortex, bioavailability of GCs is regulated *via* corticosteroid binding globulin (CBG) and corticosteroid 11β-dehydrogenase (11β-HSD) in peripheral organs (16). Because GCs are hydrophobic molecules, GCs require a transporter and CBG acts as a buffer and carrier. Neutrophil elastase induces cleavage of CBG to lead the delivery of GCs to cells. As the expression level of CBG also follow the circadian rhythm (17), this might contribute to the circadian oscillation of bioavailability of GCs. After delivery of GCs, active cortisol and inactive cortisone are interconverted by two isozymes of 11β-HSD, 11β-HSD1 and 11β-HSD2, in each organ (16). The 11β-HSD1 mainly metabolizes cortisone to cortisol, while the 11β-HSD2 converts cortisol to cortisone. Several papers reported that inflammation induced 11β-HSD1 expression in tissues *via* TNF-α in rheumatoid arthritis (RA), colitis, and chronic kidney disease, suggesting that inflammation augments effects of GCs by induction of 11β-HSD1 (18–21). Taken together, not only the hypothalamus-PVN axis but also CBG and 11β-HSD control the effects of GC *via* circadian rhythm and inflammation.

GCs exert their effects through complicated mechanisms (22). In general, GCs bind to glucocorticoid receptor (GR) in the cytoplasm (23), which induces the dimerization of GR and its translocation into the nucleus. There, GR acts as a transcription factor that promotes or suppresses the transcription of target genes by binding to specific DNA sequences known as glucocorticoid-response elements (GREs). In some cases, GR represses transcription by binding to negative GREs (nGREs). Binding of GR monomer to nGRE recruits transcriptional co-repressors, which suppresses transcriptional activation by NF-κB nearby, without direct interaction to NF-κB (22). In other case, GR monomer directly interacts with DNA-bound NF-κB and AP-1 and tethers transcriptional corepressors, without DNA binding of GR (24, 25). Transcriptional induction by GR, however, might be more important than transcriptional repression *via* nGREs and tethering. First, GCs induce the transcription of IκBα, A20, DUSP1, and GILZ, negative regulators of NF-κB and AP-1, which inhibits macrophage activation by LPS (26). Second, GC-induced transcription might snatch transcriptional coactivators and chromatin remodeling factors from the enhancers in target genes of inflammatory cytokines and reduce their transcription (2).

## Regulation of Immune-Related Gene Expression by GR

GR represses the production of inflammatory cytokines and proteins such as IL-6, C3, and TSLP by binding to nGREs and recruiting the corepressors NCOR2 and HDAC2 (27). In addition, GR induces the expression of immunosuppressive molecules such as IκBα, A20 (TNFAIP3), DUSP1, and GILZ. IκBα binds to NF-κB and blocks the activation of NF-κB (28). The A20/TAX1BP1 deubiquitinase complex inhibits the ubiquitination and degradation of RIP1 and enhances the degradation of E2 enzyme Ubc13, which suppresses NF-κB activation (28). The phosphatase DUSP1 suppresses the MAPK pathway including p38, JNK, and ERK (29, 30). DUSP1 dephosphorylates ERK and inhibits the activation of p38 and JNK, which reduces the expression of inflammatory cytokines and chemokines such as IL-1β, IL-6, GM-CSF, CCL2, CXCL1, and CXCL2. GILZ suppresses NF-κB by preventing nuclear transport of NF-κB p65 subunit and inhibits MAPK signaling by directly binding to Ras and Raf-1 (31–33). Thus, these molecules induced by GCs attenuate inflammation by inhibiting NF-κB and MAPK cascades.

Furthermore, NF-κB and GR might cooperatively control gene expression. Vollmer et al. reported that induction of DUSP-1 by GR agonist was enhanced by LPS stimulation in macrophages (34). In addition, Kadiyala et al. reported that GR and NF-κB cooperatively bound to the enhancer of the A20 locus and induced A20 expression (35). However, Rao et al. reported that increase of GR-binding sites in HeLa cells after stimulation with TNF-α and a GR agonist (~1,000 sites) was much smaller than all GR-binding sites after GR agonist stimulation (~8,700 sites) and NF-κB binding sites after TNF-α stimulation (~12,000 sites), suggesting that GR-NF-κB interaction might be partial in GR repression mechanisms (36).

On the other hand, Oh and colleagues suggested that inflammation is not critical for immunosuppressive functions of GR. They analyzed the transcription and chromatinscape of macrophages with dexamethasone (DEX) treatment before and after LPS-stimulation (26). They found that DEX treatment after LPS stimulation showed similar gene expression profile to DEX treatment before LPS stimulation. DEX treatment before and after LPS stimulation showed the upregulation of NF-κB and AP-1 inhibitors such as IκBα, A20, DUSP1, GILZ, which were critical for extinguishing inflammation. Thus, these results suggest that the induction of immunoregulatory factors by GR is important for immune regulation, independently of nGREs and tethering.

To induce transcription, GR binding to palindromic GRE might not be necessary. Schiller et al. performed ChIP-seq analysis with a human osteosarcoma cell line expressing GR with a mutation in dimerization domain and found that DNA-binding of the mutant GR was mostly overlapped with that of wild-type GR (37). It suggests that GR monomer and widespread GRE half-sites are enough for effects of GR. Sasse et al. performed global run-on sequencing (GRO-seq) in human airway epithelial cells to detect nascent RNA and found that GR repressed transcription within 10 minutes (38). By integrating the results of ChIP-seq and GRO-seq, they found that rapid repression of TNF-induced genes by GR did not require local GR-binding to canonical GREs in TNF-induced gene loci. Moreover, GR rapidly changed the accessibility of TNF-induced gene enhancers. Based on these observations, Gerber et al. proposed the squelching model that GR binding to GREs might snatch transcriptional coactivators and chromatin remodeling factors from the enhancers of TNF-induced genes and reduce their transcription of the TNF-induced genes (2). Taken together, transcriptional regulation mediated by GR is highly diversified.

## Immunosuppressive Effects of GCs

Endogenous GCs suppress inflammatory responses *via* innate immune cells and stromal cells in mouse disease models. As for the innate immune cells, GR-deficient macrophages express higher levels of inflammatory molecules, such as IL-6, TNF-α, and COX-2, through the overactivation of p38 MAPK after stimulation with LPS, leading to higher mortality (39). In contrast to the suppression of inflammatory macrophages, GCs enhance the differentiation of tissue-repair macrophages (40, 41). Galuppo et al. reported that LysM-Cre GR-deficient mice exhibited higher mortality and impaired tissue repair in a myocardial infarction model (42). In addition, Ly6C^low^ monocyte-derived macrophages in the infarcted myocardium of GR-deficient mice were reduced in number and expressed lower levels of genes related with neovascularization, collagen degradation, and scar formation. At the same time, the expression of the inflammatory chemokine CCL5 was upregulated. Thus, endogenous GCs suppress inflammatory macrophages but enhance suppressive macrophages.

Endogenous GCs suppress the maturation and function of dendritic cells (DCs). Li et al. reported that GR-deficient DCs secrete large amounts of inflammatory cytokines, such as IL-1β, IL-12, and TNF-α, which increased IFN-γ production in NK cells and caused higher mortality of GR-deficient mice (43). Elftman et al. showed that the expression of B7 and MHC class II, the maturation markers of DCs, was attenuated by GC treatment *in vitro* and *in vivo*, suggesting that GCs suppress DC maturation (44). Thus, GC-treated DCs failed to activate CD8 T cells in herpes simplex virus infection. On the other hand, DEX promotes the differentiation of IL-10-producing tolerogenic DCs (45). Hodrea et al. reported that DEX enhances phagocytosis of human DCs by inducing the expression of the apoptophagocytic genes, ADORA3 (adenosine receptor guiding macrophages to apoptotic cells), CD14, and MERTK (phagocytic receptor for apoptotic cells) (46). Therefore, endogenous and exogenous GCs suppress immunoenhancing function but enhance immunosuppressive function of DCs.

Like innate immune cells, GCs suppress cytokine production in stromal cells and alleviate colitis and asthma. Aranda et al. showed that intestinal epithelial cell-specific villin-Cre GR-deficient mice exacerbated DSS-induced colitis by increase of the neutrophil-recruiting chemokines, CXCL1, CXCL5, and CCL5 (47). Klassen et al. reported that OVA-induced allergic asthma in the lung was alleviated by DEX but that GR deficiency in the airway epithelium (SPC-Cre GR-deficient mice), but not in T cells nor DCs, canceled the suppressive effect of DEX (48). Gibbs et al. reported that GR represses the expression of the neutrophil-recruiting chemokine CXCL5 *via* nGRE in the CXCL5 promoter in an LPS-induced lung inflammation model (49). Indeed, adrenalectomized mice exhibited higher levels of CXCL5 at night, when the GC concentration is high, with a loss of circadian changes in the neutrophil accumulation in the lung. By contrast, Ince et al. reported that airway club cell-specific GR-deficient mice showed normal oscillations of LPS-induced neutrophil homing to the lung despite the loss of the circadian rhythm of CXCL5 and IL-6 (50). In addition, macrophage-specific LysM-Cre GR-deficient mice showed no diurnal change of CXCL5 and TNF-α but normal change in neutrophil count in the lung. These findings demonstrate that GCs at physiological concentrations suppress inflammation by affecting both immune cells and stromal cells. However, further studies are needed to understand how GCs control neutrophil homing to the lung. In contrast to suppression by GCs, it was also reported that DEX promoted the expression of TLR2 and soluble leukocyte protease inhibitor (SLPI), an antimicrobial molecule, in airway epithelial cells, suggesting that exogenous GCs contribute to immune response against bacterial infection (51–53). Interestingly, DEX-induced DUSP1 promoted IL-1β-driven TLR2 induction by inhibiting p38- and JNK-mediated negative feedback of TLR2 expression (52, 54, 55). Furthermore, DEX-induced DUSP1 maintained IL-1β-induced IRF1 upregulation and IRF-dependent CXCL10 expression. Thus, endogenous and exogenous GCs might support the first defense of lung epithelium but suppress excessive inflammation after infection.

GCs strongly impair the cell-mediated immunity mediated by IFN-γ-producing type-1 helper T (Th1) cells, CD8 T cells, and NK cells. Blotta et al. reported that DEX suppressed IL-12 production in human monocytes and impair IFN-γ production in NK cells (56). Moreover, Quatrini et al. reported that endogenous GCs directly suppressed the immune response of NK cells (57). GR-deficient NK cells from NK cell-specific Ncr1-Cre GR-deficient mice produced higher amounts of IFN-γ after stimulation with IL-12 and IL-18 *in vitro*. In addition, these mice exhibited increased IFN-γ production and higher lethality after the administration of LPS without any change in IL-6 or TNF-α. Furthermore, GR-deficient NK cells induced excessive inflammation and the lethality of mice with mouse cytomegalovirus (MCMV) infection, because GR deficiency decreased PD-1 expression and elevated IFN-γ production (4).

Like NK cells, endogenous GCs suppress the differentiation and IFN-γ expression of T cells. They also reduce the expression of IL-12R in T cells (58, 59). In addition, GR interacts with T-bet to inhibit its DNA-binding activity (59). Kugler et al. reported that, in a Toxoplasma infection model, T cell-specific Lck-Cre GR-deficient mice exhibited Th1 cells that had normal differentiation but also produced abnormally high levels of IFN-γ and TNF-α, leading to higher mortality (5). Thus, GCs suppress IFN-γ production by Th1 cells and thereby prevent excessive inflammation. GCs also cause dysfunctional CD8 T cells. Acharya et al. found that monocyte-macrophage lineage cells in tumors expressed the enzyme for steroidogenesis, Cyp11a1. and produced local GCs (60). In addition, exhausted CD8 T cells in tumors showed higher expressions of GR. In a MC38-OVA tumor model, GR-deficient CD8 T cells expressed higher IL-2, TNF-α, and IFN-γ; blocked tumor-growth and were less exhausted. These data suggest that GCs produced by monocyte-macrophage lineage cells promote the exhaustion of CD8 T cells and impair the immune surveillance against tumors. Taken together, endogenous GCs suppress excessive inflammation and cell-mediated immunity *via* innate and cytotoxic immune cells.

## The Circadian Rhythm of T Cell Immunity Is Controlled by Endogenous Oscillating GCs

Although GCs strongly repress inflammatory cytokine production, past studies have reported that GCs induced the expression of receptors for IL-2, IL-6, IFN-γ, GM-CSF, and TNF-α, implicating positive effects by GCs on immunity (61). In addition, Franchimont et al. reported that human blood T cells stimulated with DEX increased the transcription of genes related with cell proliferation (Mt1l, Mt1e, and Mt1b), metabolism (Sat1, Vdr), oxidation damage (Ido1), and cell surface receptors (Il1r2, Il7r) (62). IL-7 is a member of the common γ-chain cytokine family and binds to the complex of the IL-7Rα-chain and common γ-chain. IL-7 supports the development, survival, and proliferation of T cells, B cells, and innate lymphoid cells (ILCs) (63–65). In addition, IL-7R signaling protects T cells from the apoptosis induced by GCs (62). Indeed, DEX administration increased IL-7Rα expression in human T-acute lymphoblastic leukemia cells and augmented Bcl-2 expression by IL-7, which protected leukemia cells from apoptosis (66). Like IL-7Rα, GCs also induce CXCR4 expression in immune and stromal cells. GCs elevated the CXCR4 expression in T and B lymphocytes, granulocytes, and monocytes in mouse and human (67–71). Interestingly, Leigh et al. reported that GCs enhanced CXCR4 expression in human bronchial cells, whereas Carolina et al. showed that GCs reduced CXCR4 expression in lung endothelial progenitors, implying a complicated regulation of CXCR4 expression by GCs (72, 73). Thus, GC may enhance T cell immunity by inducing IL-7Rα and CXCR4.

**Figure 1:**
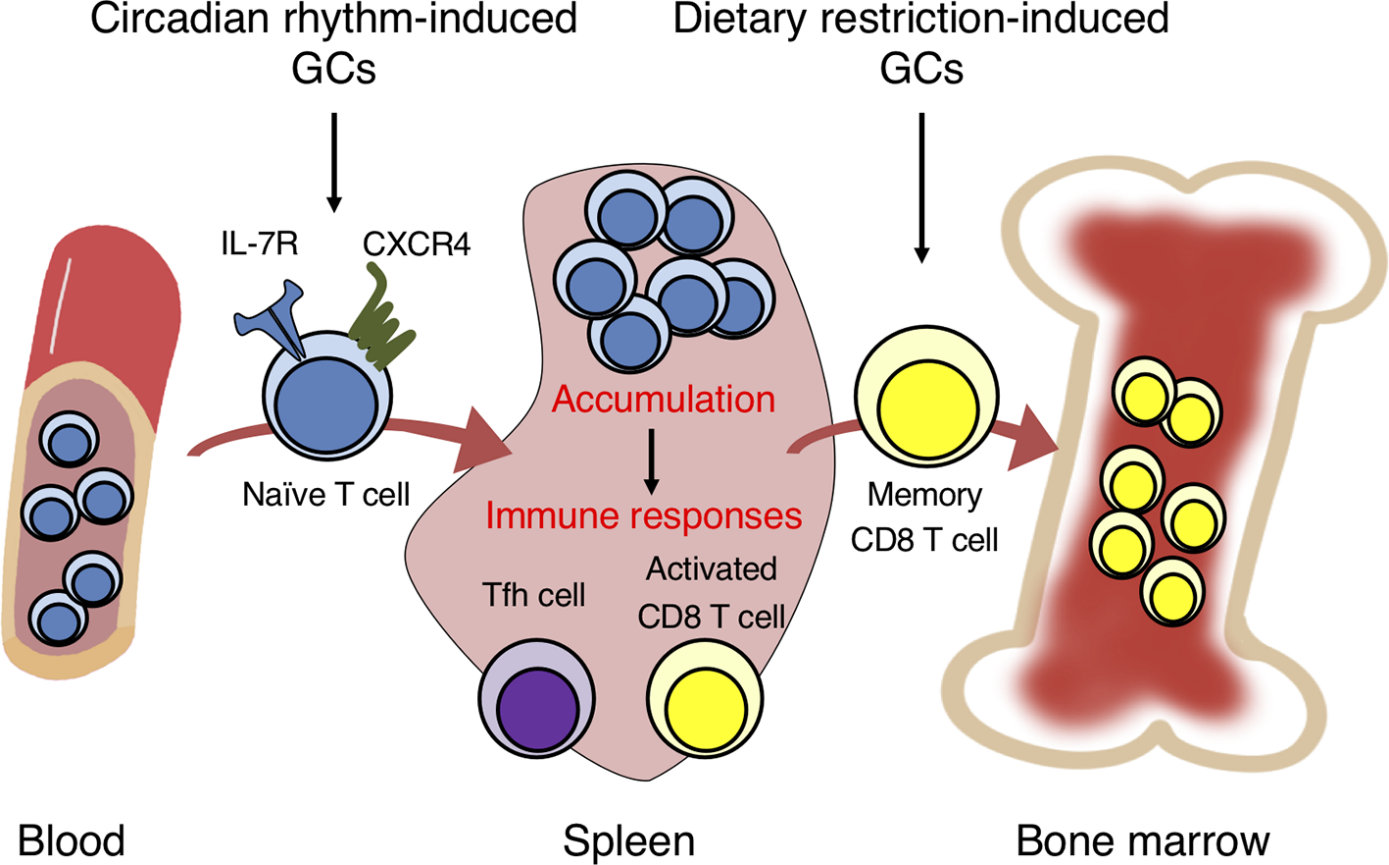
Glucocorticoids in circadian rhythm and dietary restriction induce the migration of naïve and memory T cells into the spleen and bone marrow. GCs induced by circadian rhythm promote the homing of naïve T cells into secondary lymphoid organs from peripheral blood by inducing the expression of IL-7R and CXCR4. The T cell accumulation induces strong immune responses by activated CD8 T and follicular helper T (Tfh) cells against bacterial infection and soluble antigens. GCs induced by dietary restriction trigger the egress of memory CD8 T cells from secondary lymphoid organs and homing into the bone marrow. The accumulation in the bone marrow enhances the survival and response of memory CD8 T cells.

In general, how GCs induce IL-7Rα expression on T cells is well studied. There exists a non-coding conserved sequence 1 (CNS-1) 3.6 kb upstream of the IL-7Rα promoter, the deletion of which prevented the IL-7Rα induction by GCs in mice (74, 75). The CNS-1 region contains two GRE motifs conserved between human and mouse. To investigate the biological significance of the IL-7R induction by GCs, mice harboring point mutations in the two GREs of CNS-1 (GREm mice) as well as T cell-specific CD4-Cre GR-deficient mice have been generated (7). The IL-7R expression on T cells was elevated at night and reduced at daytime in control mice, consistent with the diurnal fluctuation of GCs. Moreover, T cells in the control mice accumulated in the spleen, lymph nodes, and Peyer’s patches at nighttime, but circulated more in peripheral blood at daytime. However, the diurnal fluctuation of IL-7R expression and T cell numbers in the blood and lymphoid organs was abolished in CD4-Cre GR-deficient mice and GREm mice. This oscillation seems to be regulated partly by CXCR4, because CXCR4 expression was also induced by GCs and IL-7R. Therefore, the GC-IL-7R axis controls the diurnal oscillation of T cell distribution between the blood and lymphoid organs by regulating CXCR4 expression (**Figure 1**).

The accumulation of T cells in the lymphoid organs by GCs and IL-7R may enhance immune responses. The infection of control mice with *Listeria monocytogenes* at nighttime induced antigen-specific effector CD8 T cells more efficiently than at daytime (7). By contrast, the increase of effector CD8 T cells with nighttime infection was not observed in CD4-Cre GR-deficient or GREm mice, suggesting that the diurnal surge of GCs at night enhances the CD8 T cell response against bacterial infection. Similarly, immunization with soluble antigens at night enhanced the generation of follicular helper T (Tfh) cells, germinal center B cells, and class-switched B cells, an effect lost in the mutant mice. Furthermore, previous studies reported that GR affects the differentiation and function of helper T cell subsets (76). GCs strongly suppress the function of Th1 cells but promote the function of Th2 cells. Ramirez et al. found that primed CD4 T cells pretreated with DEX *in vitro* produced a large amount of IL-4 and IL-13 (77). Consistently, GR-deficient CD4 T cells produced lower levels of IL-4 and IL-13 in Th2-skewed culture (7). Thus, endogenous GCs promote the differentiation and function of Th2 cells. Taken together, oscillating GCs induced by the circadian rhythm have immunoenhancing effects on immunity.

Like T cells, GCs induced by the circadian rhythm enhance B cell responses. Cain et al. reported that the diurnal induction of CXCR4 in B cells was impaired in B cell-specific mb1-Cre GR-deficient mice, which attenuated B cell homing into the bone marrow (69). Additionally, B cell numbers in the blood of GR-deficient mice lost their normal diurnal change, indicating that GCs control the diurnal change of B cell recirculation between the bone marrow and blood *via* CXCR4 induction. IgG production after immunization with the T-independent antigen NP-Ficoll was impaired in GR-deficient mice, suggesting that endogenous GCs per se might promote the activation of B cells. Furthermore, it is reported that adrenergic signaling and the clock gene Bmal1 control the diurnal change of B cell recirculation between the blood and lymph nodes and that B cell retention in the lymph nodes enhances B cell responses (78, 79). Therefore, CXCR4 induction by GCs might trigger B cell homing to the lymph nodes and bone marrow and elevate the responses of B cells. Taken together, GCs induced by the circadian rhythm enhance B cell responses through the accumulation of lymphocytes in lymphoid organs and the induction of Th2 and Tfh cell differentiation (**Table 1**).

**Table 1:** A summary of the pleiotropic effects of glucocorticoids in different conditions.

	Suppression of immune responses	Enhancement of immune responses
**Exogeneous glucocorticoids**	• Suppression of inflammatory cytokine production (1–3) • Induction of lymphocyte apoptosis (3) • Suppression of function and development of Th1, NK, and CD8 T cells (4, 5, 56–60)	• Promotion of differentiation of Th2 and Th17 cells (7, 9, 77, 85, 86)
**Circadian rhythminduced glucocorticoids**	• Suppression of CXCL5 production and neutrophil recruitment in lung inflammation (49, 50)	• Induction of IL-7R and CXCR4 (7, 61, 74, 75) • Homing of T cells to lymphoid organs (7) • Enhancement of Immune response of CD8 T and Tfh cells (7)
**Dietary restrictioninduced glucocorticoids**	• Suppression of inflammatory cytokine level in serum (80)	• Migration of memory CD8 T cells into bone marrow (8) • Induction of Bcl2 expression to enhance the survival of memory CD8 T cells (8) • Enhancement of anti-cancer response by memory CD8 T cells (8)
**Stressinduced glucocorticoids**	• Suppression of IFN-γ production in Th1 and CD8 T cells (81–84) • Inhibition of CD8 T cell response against cancer and viral infection (81–84)	• Increase of IL-17 and neutrophil recruitment in sickle cell disease model (10)

References are shown in parentheses.

## GCs Under Dietary Restriction (DR) Promote Memory CD8 T Cell Response

DR contributes to longevity and reduces inflammation and cancer (80, 87). Because immune cells consume large amount of energy in inflammation, M1 macrophages and effector T cells activate glycolytic and lipogenic metabolic pathways for rapid ATP synthesis (88). Thus, the undernutrition might impair the maintenance and response of leukocytes. DR reduced the mass of thymus and spleen and induced reversible lymphopenia of T, B, and NK cells (89, 90). DR also suppressed the PI3K/Akt/mTOR signaling *via* activation of AMPK and sirtuin, which impaired the function of effector T cells and M1 macrophages (91). In addition, Yang et al. reported that serum TNF-α levels decreased with elevated cortisol under calorie restriction (CR), suggesting that CR suppresses inflammation by inducing GC production (80). On the other hand, DR enhances the cytotoxicity of CD8 T cells. Di Biase et al. reported that a fasting-mimicking diet increased common lymphoid progenitors in the bone marrow and tumor-infiltrating CD8 T cells in a breast cancer model (92). Therefore, DR promotes lymphopoiesis and cell-mediated immunity. In line with that study, it is reported that diet affects cell-mediated immune responses by inducing GCs. Collins et al. reported that GCs induced by DR enhanced the maintenance and function of memory CD8 T cells (8). DR triggered the migration of memory CD8 T cells from secondary lymphoid organs into the bone marrow, but this effect was abolished in adrenalectomized mice and T cell-specific Lck-Cre GR-deficient mice. In addition, the accumulation of memory CD8 T cells in the bone marrow by DR was impaired in CXCR4- and S1PR1-deficient mice, suggesting that GCs might induce the egress of memory CD8 T cells from secondary lymphoid organs *via* S1PR1 and drive homing to the bone marrow *via* CXCR4 (**Figure 1**). Interestingly, DR reduced mTOR signaling in memory CD8 T cells and changed gene expressions associated with heat-shock protein chaperone binding and the regulation of protein folding. It also induced amino-acid deprivation and the cellular response to rapamycin, suggesting that DR changes the metabolic state of memory CD8 T cells to quiescent. In addition, DR and DEX increased Bcl-2 expression in memory CD8 T cells. Finally, memory CD8 T cells efficiently responded to bacterial infection and tumors during DR. Together, these reports demonstrate that GCs induced by DR enhance the long-term maintenance of memory CD8 T cells, which promotes cytotoxic responses against infection and tumors.

As described above, GCs produced by monocyte-macrophage lineage cells in the tumor microenvironment suppress the function of effector CD8 T cells, whereas DR-induced GCs enhance the maintenance and response of memory CD8 T cells (8, 60). Thus, GCs seem to exert different effects on immune cells, probably by factors supplied from the microenvironment. Circadian rhythm- and DR-induced GCs trigger the homing of IL-7R-positive CD8 T cells into lymphoid organs, which supply pro-survival factors and enhance the maintenance and response of T cells. By contrast, GCs produced in the tumor microenvironment accelerate the dysfunction of IL-7R-negative effector CD8 T cells. Therefore, the supply of IL-7 from the microenvironment may determine whether the GC effects are positive or negative.

## Stress Suppresses Cell-Mediated Immunity But Enhances Inflammatory Diseases

Because stress-induced GCs suppress cell-mediated immunity, stress might exacerbate viral infection and tumor growth. Steelman et al. reported that restraint stress reduced IFN-γ-expressing CD4 T and CD8 T cells and their T-bet expression following Theiler’s murine encephalomyelitis virus (TMEV) infection (81). In addition, treatment with a GC antagonist alleviated the clinical manifestations induced by the restraint stress. Elftman et al. found that restraint stress impaired the expression of granzyme B and IFN-γ in CD8 T cells following herpes simplex virus (HSV) infection (82). However, this effect was alleviated if the infection was in T cell-specific Lck-Cre GR-deficient mice, suggesting that stress-induced GCs suppress the production of granzyme B and IFN-γ by CD8 T cells *via* GR. These findings indicate that stress exacerbates the viral infection by suppressing cell-mediated immunity *via* GCs. Like viral infection, stress caused by chronic sleep restriction, forced swimming, and abdominal surgery promoted the progression of cancers (83, 84). Hong et al. reported the relationship between perinatal stress and cell-mediated immunity (6). The perinatal exposure of fetal mice to DEX as a stress model diminished CD8 T cell response against tumors in adulthood. Thus, exposure to stress-induced GCs during pregnancy appear to cause dysfunction of anti-tumor response by CD8 T cells in offspring after birth. Overall, stress-induced GCs might impair cell-mediated immunity over the lifetime.

Although stress suppresses cell-mediated immunity, it also triggers chronic inflammation and autoimmunity. Qiu et al. reported that restraint stress enhanced tissue destruction in a DNBS-induced colitis model (93). The colitis was ameliorated in CD4-deficient mice, suggesting that helper T cell function is critical for the stress-induced tissue destruction. Arima et al. reported that wet bedding and restraint stress triggered upper gastrointestinal bleeding in mice transferred with myelin oligodendrocyte glycoprotein (MOG) peptide-primed pathogenic T cells (94). Because Th17 cells play a critical role in experimental autoimmune encephalomyelitis (EAE), the stress might enhance Th17 cell function. de Castro Kroner et al. reported that GCs elevated RAR-related orphan receptor C (RORC) expression by inhibiting IL-2 secretion in human T cells (85). In addition, GCs induced the expression of defensins and CCL20. In mouse T cells, GCs promoted the IL-23-dependent differentiation of Th17 cells *in vitro* but not if IL-6 and TGF-β were also present (9). In addition, Marchetti et al. reported that transgenic (Tg) mice expressing GR antisense RNA exhibited less severe neurological inflammation (86). Thus, GCs have the potential to enhance the function of Th17 cells. Furthermore, Xu et al. showed that restraint stress increased serum IL-17 and aged neutrophils, which exacerbated vaso-occlusive episodes *via* microbiota in a sickle cell disease model (10). Treatment with metyrapone, an inhibitor of GC production, alleviated the leukocyte recruitment and inflammation under stress. Overall, stress-induced GCs aggravate inflammation by promoting the differentiation and function of Th17 cells *via* microbiota.

Although GCs exert some anti-inflammatory effects in neutrophils, such as reduction in COX-2 and iNOS expression and superoxide release (95–97), GCs also promote the development and function of neutrophils. First, GCs enhanced the development and proliferation of neutrophils in bone marrow and induced neutrophilia in peripheral blood (98, 99). Second, GCs promote survival of neutrophils. GCs inhibited spontaneous apoptosis of human neutrophils *in vitro* (100, 101). Chang et al. reported that DEX treatment reduced the expression of Fas and caspase-8 in neutrophils (102). Furthermore, Bouterse et al. found that the expression of a pro-apoptotic factor Bak was reduced in DEX-treated neutrophils (103). Third, GCs elevated the expression of the IL-1R in neutrophils and enhanced IL-1β-triggered inflammation (104). Thus, GCs might augment tissue inflammation by enhancing the functions of neutrophils.

Stress-induced GCs control inflammation also *via* Treg cells. Harpaz et al. found that chronic variable stress reduced the number of Treg cells and increased the susceptibility to EAE (105). In addition, the administration of mifepristone, an antagonist of GCs, blocked the stress-induced exacerbation of EAE. On the other hand, it is reported that GCs also promote Treg cell function. GC stimulation upregulates the expression of Foxp3, IL-10, TGF-β, and CTLA4 (106–108). Rocamora-Reverte et al. reported that GR-deficient Treg cells failed to suppress tissue damage in a colitis model in Treg-specific Foxp3-Cre GR-deficient mice (109). Furthermore, Engler et al. reported that the onset of EAE was delayed in pregnant mice, because progesterone binding to GR increased the number of Treg cells (110). Treatment with a GC antagonist and T cell-specific Lck-Cre GR-deletion blocked the activation of Treg cells. However, it remains unknown whether stress enhances inflammation by blocking Treg cells even though GCs enhance Treg function. One possibility is that an unknown factor might block the GC-enhanced Treg function to promote inflammation under stress. Thus, because stress-induced GCs may affect both Th17 and Treg cells, it remains unclear whether GCs enhance inflammation *via* Th17 cells or suppress it *via* Treg cells. Taken together, stress-induced GCs seem to have pleiotropic effects either to attenuate cell-mediated immunity or to aggravate inflammation, depending on the cell types and the disease models (**Table 1**).

## GC Resistance in Inflammatory Diseases

GCs are well used for therapy of allergic and autoimmune diseases. GCs mitigate the symptoms in psoriasis and multiple sclerosis, whereas some patients with ulcerative colitis (UC) and asthma are refractory to treatment with GCs (111, 112). However, the mechanism of this GC resistance is unclear. One explanation is that GCs might promote survival of immune cells and enhance immune responses. As explained above, GCs have the potential to augment immune responses of Th2 cells, Th17 cells, and neutrophils, which exacerbates tissue damage. Second, some immune cells escape from the suppressive effects of GCs by reducing the amounts of GCs and GR in the cytoplasm. Ramesh et al. reported that human Th17 cells expressed multiple drug resistance 1 (MDR1), a membrane efflux pump with broad substrate specificity, which reduces the sensitivity to GCs (113). Paugh et al. found that caspase-1 activated by NLRP3 inflammasome cleaved GR (114). Third, some inflammatory cytokines such as IFN-γ, IL-17, IL-4, and TNF-α canceled the suppressive effects of GCs (115–119). These cytokines diminish GC-induced apoptosis of immune cells and blunt the repression of inflammatory cytokine production by GCs. Thus, both the enhancing effects of GCs and the cancelation of GC effects might contribute to the GC resistance in the treatment of inflammatory diseases. Manipulation of these mechanisms will facilitate to overcome the GC resistance and contribute to cure allergic and autoimmune diseases.

## Conclusions

This review summarizes the immunoenhancing and immunosuppressive effects of GCs (**Table 1**). Exogeneous and stress-induced GCs suppress the IFN-γ production and exhaustion of CD8 T cells, whereas GCs under circadian rhythm or DR enhance the maintenance and activation of naïve and memory CD8 T cells. In addition, GCs strongly suppress the function of Th1 cells and enhance the differentiation of Th2 and Th17 cells. Thus, the effects of GCs on immunity can be positive or negative depending on the tissue and cell type. The immunoenhancing effects of GCs possibly depend on the microenvironment because GCs trigger T cell homing to lymphoid organs, which supply pro-survival cytokines. Further studies are required to understand how GCs control the interaction between immune cells and the microenvironment. Moreover, the effects of GCs might depend on different GR-target genes in different cell types. To address this question, it is necessary to investigate GR-induced changes in gene transcription, DNA binding, and chromatin accessibility in immune and stromal cells. Revealing the pleiotropic effects of GCs will help understand how GCs trigger immune dysfunction and chronic inflammation and maximize the therapeutic effects of GCs in refractory allergies and autoimmune diseases.

## Author Contributions

AS wrote the first draft of the manuscript. AE and KI modified, revised, and approved the submitted version. All authors contributed to the article andapproved the submitted version.

## Funding

This work was supported by JSPS KAKENHI Grant Numbers 20K21525 (KI) and 20K16280 (AS). AS was supported by the Shimizu Foundation for Immunology and Neuroscience grant for 2016, by a grant from the Takeda Science Foundation, and by a grant from the Ichiro Kanehara Foundation for the Promotion of Medical Sciences and Medical care for 2018. AE was supported by the PhD Scholarship (Kibou Project) from Japanese Society for Immunology. KI was supported by a grant from the Naito Foundation and by a grant from the Uehara Memorial Foundation. It was also supported by the Joint Usage Research Center program of the Institute for Frontier Life and Medical Sciences, Kyoto University.

## Conflict of Interest

The authors declare that the research was conducted in the absence of any commercial or financial relationships that could be construed as a potential conflict of interest.

## Publisher’s Note

All claims expressed in this article are solely those of the authors and do not necessarily represent those of their affiliated organizations, or those of the publisher, the editors and the reviewers. Any product that may be evaluated in this article, or claim that may be made by its manufacturer, is not guaranteed or endorsed by the publisher.

## References

[1] Cain DW Cidlowski JA. Immune Regulation by Glucocorticoids. Nat Rev Immunol (2017) 17:233–47. doi: 10.1038/nri.2017.1 PMC9761406 28192415

[2] Gerber AN Newton R Sasse SK. Repression of Transcription by the Glucocorticoid Receptor: A Parsimonious Model for the Genomics Era. J Biol Chem (2021) 296:100687. doi: 10.1016/j.jbc.2021.100687 33891947 PMC8141881

[3] Herold MJ McPherson KG Reichardt HM. Glucocorticoids in T Cell Apoptosis and Function. Cell Mol Life Sci (2006) 63:60–72. doi: 10.1007/s00018-005-5390-y 16314919 PMC2792342

[4] Quatrini L Wieduwild E Escaliere B Filtjens J Chasson L Laprie C. Endogenous Glucocorticoids Control Host Resistance to Viral Infection Through the Tissue-Specific Regulation of PD-1 Expression on NK Cells. Nat Immunol (2018) 19:954–62. doi: 10.1038/s41590-018-0185-0 PMC6138242 30127438

[5] Kugler DG Mittelstadt PR Ashwell JD Sher A Jankovic D. CD4^+^ T Cells Are Trigger and Target of the Glucocorticoid Response That Prevents Lethal Immunopathology in Toxoplasma Infection. J Exp Med (2013) 210:1919–27. doi: 10.1084/jem.20122300 PMC3782051 23980098

[6] Hong JY Lim J Carvalho F Cho JY Vaidyanathan B Yu S. Medzhitov. Long-Term Programming of CD8 T Cell Immunity by Perinatal Exposure to Glucocorticoids. Cell (2020) 180:847–61. doi: 10.1016/j.cell.2020.02.018 PMC7399609 32142678

[7] Shimba A Cui GW Tani-ichi S Ogawa M Abe S Okazaki F. Glucocorticoids Drive Diurnal Oscillations in T Cell Distribution and Responses by Inducing Interleukin-7 Receptor and CXCR4. Immunity (2018) 48:286–98. doi: 10.1016/j.immuni.2018.01.004 29396162

[8] Collins N Han SJ Enamorado M Link VM Huang B Moseman EA. The Bone Marrow Protects and Optimizes Immunological Memory During Dietary Restriction. Cell (2019) 178:1088–101. doi: 10.1016/j.cell.2019.07.049 PMC6818271 31442402

[9] Zhao J Lloyd CM Noble A. Th17 Responses in Chronic Allergic Airway Inflammation Abrogate Regulatory T-Cell-Mediated Tolerance and Contribute to Airway Remodeling. Mucosal Immunol (2013) 6:335–46. doi: 10.1038/mi.2012.76 PMC4233308 22892938

[10] Xu CL Lee SK Zhang DC Frenette PS. The Gut Microbiome Regulates Psychological-Stress-Induced Inflammation. Immunity (2020) 53:417–28. doi: 10.1016/j.immuni.2020.06.025 PMC7461158 32735844

[11] Fisk AS Tam SKE Brown LA Vyazovskiy VV Bannerman DM Peirson SN. Light and Cognition: Roles for Circadian Rhythms, Sleep, and Arousal. Front Neurol (2018) 9:56. doi: 10.3389/fneur.2018.00056 29479335 PMC5811463

[12] Gjerstad JK Lightman SL Spiga F. Role of Glucocorticoid Negative Feedback in the Regulation of HPA Axis Pulsatility. Stress (2018) 21:403–16. doi: 10.1080/10253890.2018.1470238 PMC6220752 29764284

[13] Dunn AJ Swiergiel AH. The Role of Corticotropin-Releasing Factor and Noradrenaline in Stress-Related Responses, and the Inter-Relationships Between the Two Systems. Eur J Pharmacol (2008) 583:186–93. doi: 10.1016/j.ejphar.2007.11.069 PMC2661014 18281033

[14] McCall JG Al-Hasani R Siuda ER Hong DY Norris AJ Ford CP. CRH Engagement of the Locus Coeruleus Noradrenergic System Mediates Stress-Induced Anxiety. Neuron (2015) 87:605–20. doi: 10.1016/j.neuron.2015.07.002 PMC4529361 26212712

[15] Besedovsky L Born LJ Lange T. Blockade of Mineralocorticoid Receptors Enhances Naive T-Helper Cell Counts During Early Sleep in Humans. Brain Behav Immun (2012) 26:1116–21. doi: 10.1016/j.bbi.2012.07.016 22884414

[16] Perogamvros I Ray DW Trainer PJ. Regulation of Cortisol Bioavailability-Effects on Hormone Measurement and Action. Nat Rev Endocrinol (2012) 8:717–27. doi: 10.1038/nrendo.2012.134 22890008

[17] Melin J Hartung N Parra-Guillen ZP Whitaker MJ Ross RJ Kloft C. The Circadian Rhythm of Corticosteroid-Binding Globulin has Little Impact on Cortisol Exposure After Hydrocortisone Dosing. Clin Endocrinol (2019) 91:33–40. doi: 10.1111/cen.13969 30868607

[18] Nanus DE Filer AD Hughes B Fisher BA Taylor PC Stewart PM. TNFα Regulates Cortisol Metabolism *In Vivo* in Patients With Inflammatory Arthritis. Ann Rheum Dis (2015) 74:464–9. doi: 10.1136/annrheumdis-2013-203926 24385202

[19] Hardy R Rabbitt EH Filer A Emery P Hewison M Stewart PM. Local and Systemic Glucocorticoid Metabolism in Inflammatory Arthritis. Ann Rheum Dis (2008) 67:1204–10. doi: 10.1136/ard.2008.090662 PMC2564803 18420938

[20] Stegk JP Ebert B Martin HJ Maser E. Expression Profiles of Human 11β-Hydroxysteroid Dehydrogenases Type 1 and Type 2 in Inflammatory Bowel Diseases. Mol Cell Endocrinol (2009) 25:104–8. doi: 10.1016/j.mce.2008.10.030 19022342

[21] Sagmeister MS Taylor AE Fenton A Wall NA Chanouzas D Nightingale PG. Glucocorticoid Activation by 11β-Hydroxysteroid Dehydrogenase Enzymes in Relation to Inflammation and Glycaemic Control in Chronic Kidney Disease: A Cross-Sectional Study. Clin Endocrinol (2019) 90:241–9. doi: 10.1111/cen.13889 PMC6334281 30358903

[22] Hardy RS Raza K Cooper MS. Therapeutic Glucocorticoids: Mechanisms of Actions in Rheumatic Diseases. Nat Rev Rheumatol (2020) 16:133–44. doi: 10.1038/s41584-020-0371-y 32034322

[23] Vandevyver S Dejager L Libert C. Comprehensive Overview of the Structure and Regulation of the Glucocorticoid Receptor. Endocr Rev (2014) 35:671–93. doi: 10.1210/er.2014-1010 24937701

[24] Ratman D Vanden Berghe W Dejager L Libert C Tavernier J Beck IM. How Glucocorticoid Receptors Modulate the Activity of Other Transcription Factors: A Scope Beyond Tethering. Mol Cell Endocrinol (2013) 380:41–54. doi: 10.1016/j.mce.2012.12.014 23267834

[25] Li MD. Yang XY. A Retrospective on Nuclear Receptor Regulation of Inflammation: Lessons From GR and PPARs. PPPAR Res (2011) 2011:742785. doi: 10.1155/2011/742785 PMC3175381 21941526

[26] Oh KS Patel H Gottschalk RA Lee WS Baek S Fraser IDC. Sung. Anti-Inflammatory Chromatinscape Suggests Alternative Mechanisms of Glucocorticoid Receptor Action. Immunity (2017) 47:298–309. doi: 10.1016/j.immuni.2017.07.012 28801231 PMC5572836

[27] Surjit M Ganti KP Mukherji A Ye T Hua G Metzger D. Widespread Negative Response Elements Mediate Direct Repression by Agonist-Liganded Glucocorticoid Receptor. Cell (2011) 145:224–41. doi: 10.1016/j.cell.2011.03.027 21496643

[28] Shembade N Harhaj EW. Regulation of NF-κB Signaling by the A20 Deubiquitinase. Cell Mol Immunol (2012) 9:123–30. doi: 10.1038/cmi.2011.59 PMC3532050 22343828

[29] Abraham SM Clark AR. Dual-Specificity Phosphatase 1: A Critical Regulator of Innate Immune Responses. Biochem Soc Trans (2006) 34:1018–23. doi: 10.1042/bst0341018 17073741

[30] Kassel O Sancono A Kratzschmar J Kreft B Stassen M Cato ACB. Glucocorticoids Inhibit MAP Kinase *via* Increased Expression and Decreased Degradation of MKP-1. EMBO J (2001) 20:7108–16. doi: 10.1093/emboj/20.24.7108 PMC125780 11742987

[31] Cannarile L Delfino DV Adorisio S Riccardi C Ayroldi E. Implicating the Role of GILZ in Glucocorticoid Modulation of T-Cell Activation. Front Immunol (2019) 10:1823. doi: 10.3389/fimmu.2019.01823 31440237 PMC6693389

[32] Ayroldi E Migliorati G Bruscoli S Marchetti C Zollo O Cannarile L. Modulation of T-Cell Activation by the Glucocorticoid-Induced Leucine Zipper Factor *via* Inhibition of Nuclear Factor κB. Blood (2001) 98:743–53. doi: 10.1182/blood.V98.3.743 11468175

[33] Ronchetti S Migliorati G Riccardi C. GILZ as a Mediator of the Anti-Inflammatory Effects of Glucocorticoids. Front Endocrinol (2015) 6:170. doi: 10.3389/fendo.2015.00170 PMC4637413 26617572

[34] Vollmer TR Stockhausen A Zhang JZ. Anti-Inflammatory Effects of Mapracorat, a Novel Selective Glucocorticoid Receptor Agonist, Is Partially Mediated by MAP Kinase Phosphatase-1 (MKP-1). J Biol Chem (2012) 287:35212–21. doi: 10.1074/jbc.M112.400671 PMC3471745 22898817

[35] Kadiyala V Sasse SK Altonsy MO Berman R Chu HW Phang TL. Cistrome-Based Cooperation Between Airway Epithelial Glucocorticoid Receptor and NF-κB Orchestrates Anti-Inflammatory Effects. J Biol Chem (2016) 291:12673–87. doi: 10.1074/jbc.M116.721217 PMC4933445 27076634

[36] Rao NAS McCalman MT Moulos P Francoijs KJ Chatziioannou A Kolisis FN. Coactivation of GR and NFκB Alters the Repertoire of Their Binding Sites and Target Genes. Genome Res (2011) 21:1404–16. doi: 10.1101/gr.118042.110 PMC3166826 21750107

[37] Schiller BJ Chodankar R Watson LC Stallcup MR Yamamoto KR. Glucocorticoid Receptor Binds Half Sites as a Monomer and Regulates Specific Target Genes. Genome Biol (2014) 15:418. doi: 10.1186/s13059-014-0418-y 25085117 PMC4149261

[38] Sasse SK Gruca M Allen MA Kadiyala V Song TY Gally F. Nascent Transcript Analysis of Glucocorticoid Crosstalk With TNF Defines Primary and Cooperative Inflammatory Repression. Genome Res (2019) 29:1753–65. doi: 10.1101/gr.248187.119 PMC6836729 31519741

[39] Bhattacharyya S Brown DE Brewer JA Vogt SK Muglia LJ. Macrophage Glucocorticoid Receptors Regulate Toll-Like Receptor 4-Mediated Inflammatory Responses by Selective Inhibition of P38 MAP Kinase. Blood (2007) 109:4313–9. doi: 10.1182/blood-2006-10-048215 PMC1885507 17255352

[40] Desgeorges T Caratti G Mounier R Tuckermann J Chazaud B. Glucocorticoids Shape Macrophage Phenotype for Tissue Repair. Front Immunol (2019) 10:1591. doi: 10.3389/fimmu.2019.01591 31354730 PMC6632423

[41] Arora S Dev K Agarwal B Das P Syed MA. Macrophages: Their Role, Activation and Polarization in Pulmonary Diseases. Immunobiology (2018) 223:383–96. doi: 10.1016/j.imbio.2017.11.001 PMC7114886 29146235

[42] Galuppo P Vettorazzi S Hovelmann J Scholz CJ Tuckermann JP Bauersachs J. The Glucocorticoid Receptor in Monocyte-Derived Macrophages Is Critical for Cardiac Infarct Repair and Remodeling. FASEB J (2017) 31:5122–32. doi: 10.1096/fj.201700317R PMC5636710 28768721

[43] Li CYC Munitic I Mittelstadt PR Castro E Ashwell JD. Suppression of Dendritic Cell-Derived IL-12 by Endogenous Glucocorticoids Is Protective in LPS-Induced Sepsis. PloS Biol (2015) 13:e1002269. doi: 10.1371/journal.pbio.1002269 26440998 PMC4595142

[44] Elftman MD Norbury CC Bonneau RH Truckenmiller ME. Corticosterone Impairs Dendritic Cell Maturation and Function. Immunology (2007) 122:279–90. doi: 10.1111/j.1365-2567.2007.02637.x PMC2265998 17848165

[45] Chamorro S Garcia-Vallejo JJ Unger WWJ Fernandes RJ Bruijns SCM Laban S. TLR Triggering on Tolerogenic Dendritic Cells Results in TLR2 Up-Regulation and a Reduced Proinflammatory Immune Program. J Immunol (2009) 183:2984–94. doi: 10.4049/jimmunol.0801155 19648269

[46] Hodrea J Majai G Doro Z Zahuczky G Pap A Rajnavolgyi E. The Glucocorticoid Dexamethasone Programs Human Dendritic Cells for Enhanced Phagocytosis of Apoptotic Neutrophils and Inflammatory Response. J Leukoc Biol (2012) 91:127–36. doi: 10.1189/jlb.0511243 22028334

[47] Muzzi C Watanabe N Twomey E Meers GK Reichardt HM Bohnenberger H. The Glucocorticoid Receptor in Intestinal Epithelial Cells Alleviates Colitis and Associated Colorectal Cancer in Mice. Cell Mol Gastroenterol Hepatol (2021) 11:1505–18. doi: 10.1016/j.jcmgh.2020.12.006 PMC8039723 33316454

[48] Klassen C Karabinskaya A Dejager L Vettorazzi S Van Moorleghem J Luhder F. Airway Epithelial Cells Are Crucial Targets of Glucocorticoids in a Mouse Model of Allergic Asthma. J Immunol (2017) 199:48–61. doi: 10.4049/jimmunol.1601691 28515280

[49] Gibbs J Ince L Matthews L Mei JJ Bell T Yang N. An Epithelial Circadian Clock Controls Pulmonary Inflammation and Glucocorticoid Action. Nat Med (2014) 20:919–26. doi: 10.1038/nm.3599 PMC4268501 25064128

[50] Ince LM Zhang ZG Beesley S Vonslow RM Saer B Matthews LC. Circadian Variation in Pulmonary Inflammatory Responses Is Independent of Rhythmic Glucocorticoid Signaling in Airway Epithelial Cells. FASEB J (2019) 33:126–39. doi: 10.1096/fj.201800026RR PMC6355062 29965797

[51] Silverstein R Johnson DC. Endogenous Versus Exogenous Glucocorticoid Responses to Experimental Bacterial Sepsis. J Leukoc Biol (2003) 73:417–27. doi: 10.1189/jlb.0702379 12660216

[52] Imasato A Desbois-Mouthon C Han JH Kai H Cato ACB Akira S. Inhibition of P38 MAPK by Glucocorticoids *via* Induction of MAPK Phosphatase-1 Enhances Nontypeable Haemophilus Influenzae-Induced Expression of Toll-Like Receptor 2. J Biol Chem (2002) 277:47444–50. doi: 10.1074/jbc.M208140200 12356755

[53] Abbinantenissen JM Simpson LG Leikauf GD. Corticosteroids Increase Secretory Leucocyte Protease Inhibitor Transcript Levels in Airway Epitherial Cells. Am J Physiol (1995) 268:601–6. doi: 10.1152/ajplung.1995.268.4.L601 7733301

[54] Sakai A Han JH Cato ACB Akira S Li JD. Glucocorticoids Synergize With IL-1β to Induce TLR2 Expression *via* MAP Kinase Phosphatase-1-Dependent Dual Inhibition of MAPK JNK and P38 in Epithelial Cells. BMC Mol Biol (2004) 5:2. doi: 10.1186/14712199-5-2 15125785 PMC419700

[55] Shah S King EM Mostafa MM Altonsy MO Newton R. DUSP1 Maintains IRF1 and Leads to Increased Expression of IRF1-Dependent Genes: A Mechanism Promoting Glucocorticoid Insensitivity. J Biol Chem (2016) 291:21802–16. doi: 10.1074/jbc.M116.728964 PMC5076847 27551049

[56] Blotta MH DeKruyff RH Umetsu DT. Corticosteroids Inhibit IL-12 Production in Human Monocytes and Enhance Their Capacity to Induce IL-4 Synthesis in CD4^+^ Lymphocytes. J Immunol (1997) 158:5589–95. 9190905

[57] Quatrini L Wieduwild E Guia S Bernat C Glaichenhaus N Vivier E. Host Resistance to Endotoxic Shock Requires the Neuroendocrine Regulation of Group 1 Innate Lymphoid Cells. J Exp Med (2017) 214:3531–41. doi: 10.1084/jem.20171048 PMC5716043 29141867

[58] Franchimont D Galon J Gadina M Visconti R Zhou YJ Aringer M. Inhibition of Th1 Immune Response by Glucocorticoids, Dexamethasone Selectively Inhibits IL-12-Induced Stat4 Phosphorylation in T Lymphocytes. J Immunol (2000) 164:1768–74. doi: 10.4049/jimmunol.164.4.1768 10657623

[59] Liberman AC Refojo D Druker J Toscano M Rein T Holsboer F. The Activated Glucocorticoid Receptor Inhibits the Transcription Factor T-Bet by Direct Protein-Protein Interaction. FASEB J (2007) 21:1177–88. doi: 10.1096/fj.06-7452com 17215482

[60] Acharya N Madi A Zhang HY Klapholz M Escobar G Dulberg S. Endogenous Glucocorticoid Signaling Regulates CD8^+^ T Cell Differentiation and Development of Dysfunction in the Tumor Microenvironment. Immunity (2020) 53:658–71. doi: 10.1016/j.immuni.2020.08.005 PMC7682805 32937153

[61] Wiegers GJ Reul J. Induction of Cytokine Receptors by Glucocorticoids: Functional and Pathological Significance. Trends Pharmacol Sci (1998) 19:317–21. doi: 10.1016/s0165-6147(98)01229-2 9745359

[62] Franchimont D Galon J Vacchio MS Fan S Visconti R Frucht DM. Positive Effects of Glucocorticoids on T Cell Function by Up-Regulation of IL-7 Receptor α. J Immunol (2002) 168:2212–8. doi: 10.4049/jimmunol.168.5.2212 11859107

[63] Mazzucchelli R Durum SK. Interleukin-7 Receptor Expression: Intelligent Design. Nat Rev Immunol (2007) 7:144–54. doi: 10.1038/nri2023 17259970

[64] Tani-ichi S Shimba A Wagatsuma K Miyachi H Kitano S Imai K. Interleukin-7 Receptor Controls Development and Maturation of Late Stages of Thymocyte Subpopulations. Proc Natl Acad Sci USA (2013) 110:612–7. doi: 10.1073/pnas.1219242110 PMC3545802 23267098

[65] Diefenbach A Colonna M Koyasu S. Development, Differentiation, and Diversity of Innate Lymphoid Cells. Immunity (2014) 41:354–65. doi: 10.1016/j.immuni.2014.09.005 PMC4171710 25238093

[66] Meyer LK Huang BJ Delgado-Martin C Roy RP Hechmer A Wandler AM. Glucocorticoids Paradoxically Facilitate Steroid Resistance in T Cell Acute Lymphoblastic Leukemias and Thymocytes. J Clin Invest (2020) 130:863–76. doi: 10.1172/jci130189 PMC6994137 31687977

[67] Dimitrov S Benedict C Heutling D Westermann J Born J Lange T. Cortisol and Epinephrine Control Opposing Circadian Rhythms in T Cell Subsets. Blood (2009) 113:5134–43. doi: 10.1182/blood-2008-11-190769 PMC2686184 19293427

[68] Besedovsky L Born J Lange T. Endogenous Glucocorticoid Receptor Signaling Drives Rhythmic Changes in Human T-Cell Subset Numbers and the Expression of the Chemokine Receptor CXCR4. FASEB J (2014) 28:67–75. doi: 10.1096/fj.13-237958 24051033

[69] Cain DW Bortner CD Diaz-Jimenez D Petrillo MG Gruver-Yates A Cidlowski JA. Murine Glucocorticoid Receptors Orchestrate B Cell Migration Selectively Between Bone Marrow and Blood. J Immunol (2020) 205:619–29. doi: 10.4049/jimmunol.1901135 PMC7369236 32571841

[70] Nagase H Miyamasu M Yamaguchi M Kawasaki H Ohta K Yamamoto K. Glucocorticoids Preferentially Upregulate Functional CXCR4 Expression in Eosinophils. J Allergy Clin Immunol (2000) 106:1132–9. doi: 10.1067/mai.2000.110923 11112897

[71] Heideveld E Hampton-O’Neil LA Cross SJ van Alphen FPJ van den Biggerlaar M Toye AM. Glucocorticoids Induce Differentiation of Monocytes Towards Macrophages That Share Functional and Phenotypical Aspects With Erythroblastic Island Macrophages. Haematological (2018) 103:395–405. doi: 10.3324/haematol.2017.179341 PMC5830394 29284682

[72] Leigh R Mostafa MM King EM CRider CF Shah S Dumonceaux C. An Inhaled Dose of Budesonide Induces Genes Involved in Transcription and Signaling in the Human Airways: Enhancement of Anti- and Proinflammatory Effector Genes. Pharmacol Res Perspectl (2016) 4:e00243. doi: 10.1002/prp2.243 PMC5242176 28116096

[73] Carolina E Kato T Khanh VC Moriguchi K Yamashita T Takeuchi K. Glucocorticoid Impaired the Wound Healing Ability of Endothelial Progenitor Cells by Reducing the Expression of CXCR4 in the PGE2 Pathway. Front Med (2018) 5:276. doi: 10.3389/fmed.2018.00276 PMC6173212 30324106

[74] Lee HC Shibata H Ogawa S Maki K Ikuta K. Transcriptional Regulation of the Mouse IL-7 Receptor α Promoter by Glucocorticoid Receptor. J Immunol (2005) 174:7800–6. doi: 10.4049/jimmunol.174.12.7800 15944284

[75] Abe A Tani-ichi S Shitara S Cui G Yamada H Miyachi H. An Enhancer of the IL-7 Receptor α-Chain Locus Controls IL-7 Receptor Expression and Maintenance of Peripheral T Cells. J Immunol (2015) 195:3129–38. doi: 10.4049/jimmunol.1302447 26336149

[76] Elenkov IJ. Glucocorticoids and the Th1/Th2 Balance. Ann NY Acad Sci (2004) 1024:138–46. doi: 10.1196/annals.1321.010 15265778

[77] Ramirez F Fowell DJ Puklavec M Simmonds S Mason D. Glucocorticoids Promote a Th2 Cytokine Response by CD4^+^ T Cells *In Vitro* . J Immunol (1996) 156:2406–12. 8786298

[78] Suzuki K Hayano Y Nakai A Furuta F Noda M. Adrenergic Control of the Adaptive Immune Response by Diurnal Lymphocyte Recirculation Through Lymph Nodes. J Exp Med (2016) 213:2567–74. doi: 10.1084/jem.20160723 PMC5110024 27799619

[79] Druzd D Matveeva O Ince L Harrison U He WY Schmal C. Lymphocyte Circadian Clocks Control Lymph Node Trafficking and Adaptive Immune Responses. Immunity (2017) 46:120–32. doi: 10.1016/j.immuni.2016.12.011 PMC5263259 28087238

[80] Yang L Licastro D Cava E Veronese N Spelta F Rizza W. Long-Term Calorie Restriction Enhances Cellular Quality-Control Processes in Human Skeletal Muscle. Cell Rep (2016) 14:422–8. doi: 10.1016/j.celrep.2015.12.042 26774472

[81] Steelman AJ Dean DD Young CR Smith R Prentice TW Meagher MW. Restraint Stress Modulates Virus Specific Adaptive Immunity During Acute Theiler’s Virus Infection. Brain Behav Immun (2009) 23:830–43. doi: 10.1016/j.bbi.2009.03.010 PMC2710426 19348911

[82] Elftman MD Hunzeker JT Mellinger JC Bonneau RH Norbury CC Truckenmiller ME. Stress-Induced Glucocorticoids at the Eliest Stages of Herpes Simplex Virus-1 Infection Suppress Subsequent Antiviral Immunity, Implicating Impaired Dendritic Cell Function. J Immunol (2010) 184:1867–75. doi: 10.4049/jimmunol.0902469 PMC3701455 20089700

[83] De Lorenzo BHP Brito R Leal TP Garcia NP dos Santos RMM Alvares-Saraiva AM. Chronic Sleep Restriction Impairs the Antitumor Immune Response in Mice. Neuroimmunomodulation (2018) 25:59–67. doi: 10.1159/000490352 30007965

[84] Ben-Eliyahu S Page GG Yirmiya R Shakhar G. Evidence That Stress and Surgical Interventions Promote Tumor Development by Suppressing Natural Killer Cell Activity. Int J Cancer (1999) 80:880–8. doi: 10.1002/(sici)1097-0215(19990315)80:6<880::aid-ijc14>3.0.co;2-y 10074922

[85] de Castro Kroner J Knoke K Kofler DM Steiger J Fabri M. Glucocorticoids Promote Intrinsic Human T_H_17 Differentiation. J Allergy Clin Immunol (2018) 142:1669–73. doi: 10.1016/j.jaci.2018.07.019 30092286

[86] Marchetti B Morale MC Testa N Tirolo C Caniglia S Amor S. Stress, the Immune System and Vulnerability to Degenerative Disorders of the Central Nervous System in Transgenic Mice Expressing Glucocorticoid Receptor Antisense RNA. Brain Res Rev (2001) 37:259–72. doi: 10.1016/s0165-0173(01)00130-8 11744091

[87] Pistollato F Forbes-Hernandez TY Iglesias RC Ruiz R Elexpuru-Zabaleta M Dominguez I. Effects of Caloric Restriction on Immunosurveillance, Microbiota and Cancer Cell Phenotype: Possible Implications for Cancer Treatment. Semin Cancer Biol (2020) 30:S1104–579X(20)30255-8. doi: 10.1016/j.semcancer.2020.11.017 33271317

[88] Childs CE Calder PC Miles EA. Diet and Immune Function. Nutrients (2019) 11:1933. doi: 10.3390/nu11081933 PMC6723551 31426423

[89] Xu DL Hu XK Tian YF. Effect of Temperature and Food Restriction on Immune Function in Striped Hamsters (*Cricetulus Barabensis*). J Exp Biol (2017) 220:2187–95. doi: 10.1242/jeb.153601 28381582

[90] Contreras NA Fontana L Tosti V Nikolich-Zugich J. Calorie Restriction Induces Reversible Lymphopenia and Lymphoid Organ Atrophy Due to Cell Redistribution. Geroscience (2018) 40:279–91. doi: 10.1007/s11357-018-0022-2 PMC6060198 29804201

[91] Okawa T Nagai M Hase K. Dietary Intervention Impacts Immune Cell Functions and Dynamics by Inducing Metabolic Rewiring. Front Immunol (2021) 11:623989. doi: 10.3389/fimmu.2020.623989 33613560 PMC7890027

[92] Di Biase S Lee C Brandhorst S Manes B Buono R Cheng CW. Fasting-Mimicking Diet Reduces HO-1 to Promote T Cell-Mediated Tumor Cytotoxicity. Cancer Cell (2016) 30:136–46. doi: 10.1016/j.ccell.2016.06.005 PMC5388544 27411588

[93] Qiu BS Vallance BA Blennerhassett PA Collins SM. The Role of CD4^+^ Lymphocytes in the Susceptibility of Mice to Stress-Induced Reactivation of Experimental Colitis. Nat Med (1999) 5:1178–82. doi: 10.1038/13503 10502822

[94] Arima Y Ohki T Nishikawa N Higuchi K Ota M Tanaka Y. Brain Micro-Inflammation at Specific Vessels Dysregulates Organ-Homeostasis *via* the Activation of a New Neural Circuit. Elife (2017) 6:e25517. doi: 10.7554/eLife.25517 28809157 PMC5557598

[95] Ignacchiti MDC Sesti-Costa R Marchi LF Chedraoui-Silva S Mantovani B. Effect of Academic Psychological Stress in Post-Graduate Students: The Modulatory Role of Cortisol on Superoxide Release by Neutrophils. Stress (2011) 14:290–300. doi: 10.3109/10253890.2010.545459 21443430

[96] Satoh S Oishi K Iwagaki A Senba M Akaike T Akiyama M. Dexamethasone Impairs Pulmonary Defence Against *Pseudomonas Aeruginosa* Through Suppressing iNOS Gene Expression and Peroxynitrite Production in Mice. Clin Exp Immunol (2001) 126:266–73. doi: 10.1046/j.1365-2249.2001.01656.x PMC1906189 11703370

[97] Maloney CG Kutchera WA Albertine KH McIntyre TM Prescott SM Zimmerman GA. Inflammatory Agonists Induce Cyclooxygenase Type 2 Expression by Human Neutrophils. J Immunol (1998) 160:1402–10. 9570560

[98] Trottier MD Newsted MM King LE Fraker PJ. Natural Glucocorticoids Induce Expansion of All Developmental Stages of Murine Bone Marrow Granulocytes Without Inhibiting Function. Proc Natl Acad Sci USA (2008) 105:2028–33. doi: 10.1073/pnas.0712003105 PMC2538876 18250324

[99] Mishler JM Emerson PM. Development of Neutrophilia by Serially Increasing Doses of Dexamethasone. Br J Haematol (1977) 36:249–57. doi: 10.1111/j.1365-2141.1977.tb00646.x 871436

[100] Saffar AS Dragon S Ezzati P Shan L Gounni AS. Phosphatidylinositol 3-Kinase and P38 Mitogen-Activated Protein Kinase Regulate Induction of Mcl-1 and Survival in Glucocorticoid-Treated Human Neutrophils. J Allergy Clin Immunol (2008) 121:492–8. doi: 10.1016/j.jaci.2007.10.003 18036649

[101] Cox G. Glucocorticoid Treatment Inhibits Apoptosis in Human Neutrophils. Separation of Survival and Activation Outcomes. J Immunol (1995) 154:14719–4725. 7722324

[102] Chang LC Madsen SA Toelboell T Weber PSD Burton JL. Effects of Glucocorticoids on Fas Gene Expression in Bovine Blood Neutrophils. J Endocrinol (2004) 183:569–83. doi: 10.1677/joe.1.05822 15590983

[103] Madsen-Bouterse SA Rosa GJM Burton JL. Glucocorticoid Modulation of Bcl-2 Family Members A1 and Bak During Delayed Spontaneous Apoptosis of Bovine Blood Neutrophils. Endocrinology (2006) 147:3826–34. doi: 10.1210/en.2006-0142 16675521

[104] Shieh JH Peterson RHF Moore MAS. Cytokines and Dexamethasone Modulation of IL-1 Receptors on Human Neutrophils *In Vitro* . J Immunol (1993) 150:3515–24. 8468485

[105] Harpaz I Abutbul S Nemirovsky A Gal R Cohen H Monsonego A. Chronic Exposure to Stress Predisposes to Higher Autoimmune Susceptibility in C57BL/6 Mice: Glucocorticoids as a Double-Edged Sword. Eur J Immunol (2013) 43:758–69. doi: 10.1002/eji.201242613 23255172

[106] Karagiannidis C Akdis M Holopainen P Woolley NJ Hense G Ruckert B. Glucocorticoids Upregulate FOXP3 Expression and Regulatory T Cells in Asthma. J Allergy Clin Immunol (2004) 114:1425–33. doi: 10.1016/j.jaci.2004.07.014 15577848

[107] Ugor E Prenek L Pap R Berta G Ernszt D Najbauer J. Glucocorticoid Hormone Treatment Enhances the Cytokine Production of Regulatory T Cells by Upregulation of Foxp3 Expression. Immunobiology (2018) 223:422–31. doi: 10.1016/j.imbio.2017.10.010 29223294

[108] Chen X Murakami T Oppenheim JJ Howard DMZ. Differential Response of Murine CD4^+^CD25^+^ and CD4^+^CD25^–^ T Cells to Dexamethasone-Induced Cell Death. Eur J Immunol (2004) 34:859–69. doi: 10.1002/eji.200324506 14991616

[109] Rocamora-Reverte L Tuzlak S von Raffay L Tisch M Fiegl H Drach M. Glucocorticoid Receptor-Deficient Foxp3^+^ Regulatory T Cells Fail to Control Experimental Inflammatory Bowel Disease. Front Immunol (2019) 10:47. doi: 10.3389/fimmu.2019.0047 30936873 PMC6431616

[110] Engler JB Kursawe N Solano ME Patas K Wehrmann S Heckmann N. Glucocorticoid Receptor in T Cells Mediates Protection From Autoimmunity in Pregnancy. Proc Natl Acad Sci USA (2017) 114:181–90. doi: 10.1073/pnas.1617115114 PMC5240705 28049829

[111] Banuelos J Cao Y Shin SC Lu NZ. Immunopathology Alters Th17 Cell Glucocorticoid Sensitivity. Allergy (2017) 72:331–41. doi: 10.1111/all.13051 PMC5315659 27646878

[112] Leung DYM Bloom JW. Update on Glucocorticoid Action and Resistance. J Allergy Clin Inmmunol (2003) 111:3–23. doi: 10.1067/mai.2003.97 12532089

[113] Ramesh R Kozhaya L McKevitt K Djuretic IM Carlson TJ Quintero MA. Pro-Inflammatory Human Th17 Cells Selectively Express P-Glycoprotein and Are Refractory to Glucocorticoids. J Exp Med (2014) 211:89–104. doi: 10.1084/jem.20130301 24395888 PMC3892977

[114] Paugh SW Bonten EJ Savic D Ramsey LB Thierfelder WE Gurung P. NALP3 Inflammasome Upregulation and CASP1 Cleavage of the Glucocorticoid Receptor Cause Glucocorticoid Resistance in Leukemia Cells. Nat Genet (2015) 47:607–14. doi: 10.1038/ng.3283 PMC4449308 25938942

[115] Britt RD Thompson MA Sasse S Pabelick CM Gerber AN Prakash YS. Th1 Cytokines TNF-α and IFN-γ Promote Corticosteroid Resistance in Developing Human Airway Smooth Muscle. Am J Physiol Lung Cell Mol Physiol (2019) 316:L71–81. doi: 10.1152/ajplung.00547.2017 PMC6383501 30335498

[116] Chambers ES Nanzer AM Pfeffer PE Richards DF Timms PM Martineau AR. Distinct Endotypes of Steroid-Resistant Asthma Characterized by IL-17A^high^ and IFN-γ^High^ Immunophenotypes: Potential Benefits of Calcitriol. J Allergy Clim Immunol (2015) 136:628–37. doi: 10.1016/j.jaci.2015.01.026 PMC4559139 25772594

[117] Nguyen TH Maltby S Tay HL Eyers F Foster PS Yang M. Identification of IFN-γ and IL-27 as Critical Regulators of Respiratory Syncytial Virus-Induced Exacerbation of Allergic Airways Disease in a Mouse Model. J Immunol (2018) 200:237–47. doi: 10.4049/jimmunol.1601950 29167232

[118] Pazdrak K Straub C Maroto R Stafford S White WI Calhoun WJ. Cytokine-Induced Glucocorticoid Resistance From Eosinophil Activation: Protein Phosphatase 5 Modulation of Glucocorticoid Receptor Phosphorylation and Signaling. J Immunol (2016) 197:3782–91. doi: 10.4049/jimmunol.1601029 PMC5098561 27742828

[119] Zijlstra GJ ten Hacken NHT Hoffmann RF van Oosterhout AJM Heijink IH. Interleukin-17A Induces Glucocorticoid Insensitivity in Human Bronchial Epithelial Cells. Eur Respir J (2012) 39:439–45. doi: 10.1183/09031936.0001791 21828034

